# Repaired Flexor Tendon Excursion with Half a Fist of True Active Movement Versus Full Fist Place and Hold in the Awake Patient

**DOI:** 10.1097/GOX.0000000000002074

**Published:** 2019-04-25

**Authors:** Clifton Meals, Donald Lalonde, Gilles Candelier

**Affiliations:** From the * Orthopedics and Hand Surgery, Emory University Department of Orthopedics, Atlanta, Ga.; † Dalhousie University Division of Plastic and Reconstructive Surgery, Saint John, NB, Canada; ‡ Centre de la Main HP St Martin, Caen CEDEX, France.

## Abstract

Supplemental Digital Content is available in the text.

Wide awake flexor tendon repair permits observation of freshly repaired tendons moving actively and passively in comfortable tourniquet free unsedated patients. We present videos of true active flexion versus full fist place and hold (FFPH) immediately after tendon repair so the viewer can see how the tendons move when we simulate postoperative rehabilitation in the awake patient.

The videos demonstrate that half a fist of true active movement provides 5–15 mm of smooth normal gliding of repaired profundus tendons in 10 fingers and 1 flexor pollicis longus in a thumb in 10 patients (**See** video, Supplemental Digital Content 1, which displays 4 fingers showing a range of 5–10 mm of profundus repair glide. Buckle and jerk with FFPH simulation is clearly shown in one of the tendons. This video is available in the “Related Videos” section of the Full-Text article at PRSGlobalOpen.com or at http://links.lww.com/PRSGO/A973; **see** video, Supplemental Digital Content 2, which displays 3 fingers showing a range of 6–11 mm of profundus repair glide. This video is available in the “Related Videos” section of the Full-Text article at PRSGlobalOpen.com or at http://links.lww.com/PRSGO/A974; **see** video, Supplemental Digital Content 3, which displays 2 fingers showing 14 and 15 mm of FDP repair glide. One thumb showing 12 mm of FPL repair glide. This video is available in the “Related Videos” section of the Full-Text article at PRSGlobalOpen.com or at http://links.lww.com/PRSGO/A975).

**Video Graphic 1.**
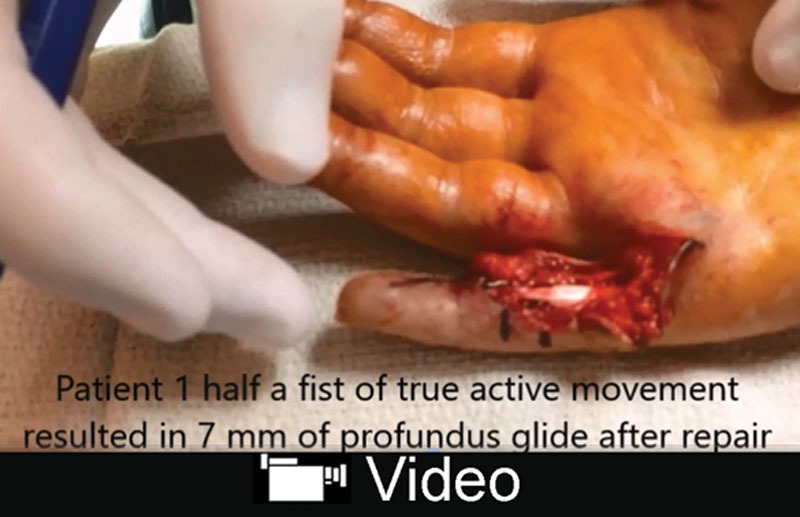
See video, Supplemental Digital Content 1, which displays 4 fingers showing a range of 5–10 mm of profundus repair glide. Buckle and jerk with FFPH simulation is clearly shown in one of the tendons. Two of the fingers had A4 vented and one of the fingers had both A3 and A4 vented. None of the fingers had clinically significant bowstringing with full fist active movement during or after the surgery. This video is available in the “Related Videos” section of the Full-Text article at PRSGlobalOpen.com or at http://links.lww.com/PRSGO/A973.

**Video Graphic 2.**
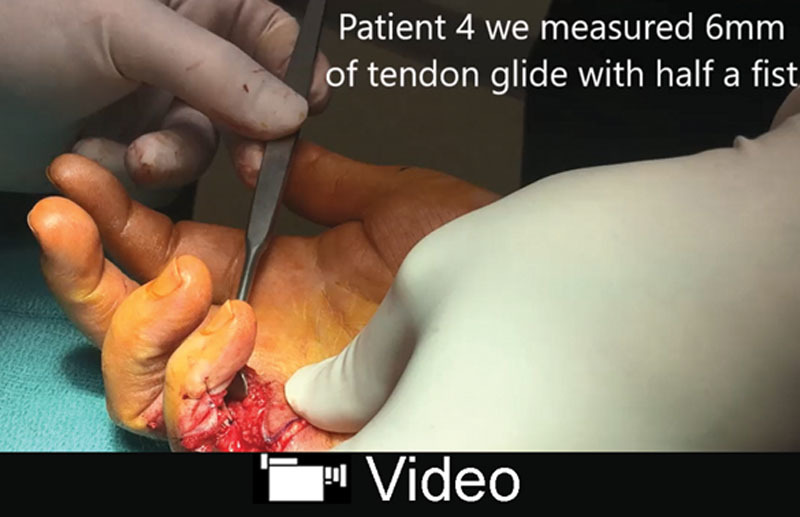
See video, Supplemental Digital Content 2, which displays 3 fingers showing a range of 6–11 mm of profundus repair glide. One of the fingers had A3 + A4 vented. None of the fingers had clinically significant bowstringing with full fist active movement during or after the surgery. This video is available in the “Related Videos” section of the Full-Text article at PRSGlobalOpen.com or at http://links.lww.com/PRSGO/A974.

**Video Graphic 3.**
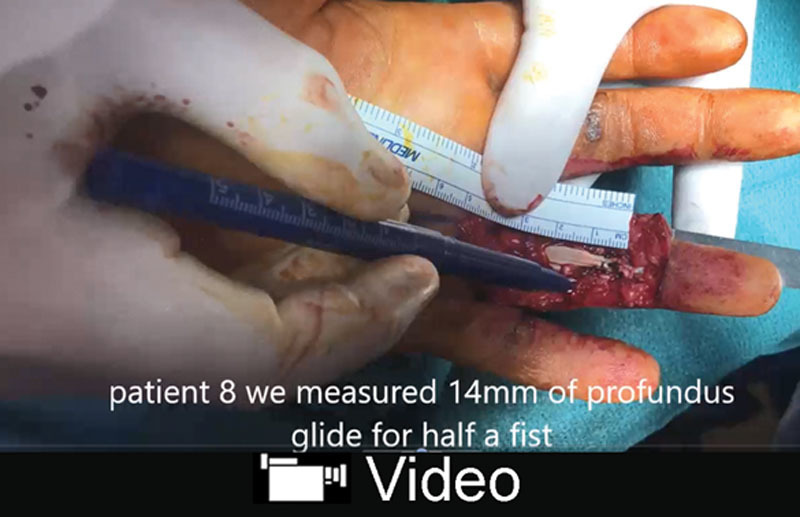
See video, Supplemental Digital Content 3, which displays 2 fingers showing 14 and 15 mm of FDP repair glide. One thumb showing 12 mm of FPL repair glide. One of the fingers had A3 + A4 vented. The other finger had A4 vented. None of the fingers had clinically significant bowstringing with full fist active movement during or after the surgery. This video is available in the “Related Videos” section of the Full-Text article at PRSGlobalOpen.com or at http://links.lww.com/PRSGO/A975.

Five to 15 mm with half a fist is sufficient to keep the tendons gliding while they heal. We know from the work of Jin Bo Tang that the last half of a full fist is where the greatest risk of rupture exists because of increased friction and work of flexion.^1^

After flexor tendon repair, popular forms postoperative rehabilitation are (1) true active movement^2^; (2) (FFPH)^3^; and (3) Kleinert rubber band passive flexion with active extension.^4^ FFPH involves passive flexion of the fingers into a full fist followed by the patient actively holding the flexed position. It is assumed that passively flexing the fingers results in full excursion of the tendons. FFPH simulation in the wide awake patient shows us that passive finger flexion does not always result in full tendon flexion. The tendon can stop gliding and buckle part way through full passive fist flexion (**Supplemental Digital Content 1**). When the patient is then asked to hold the finger in full flexion, the tendon jerks into full flexion from its passively buckled position. We feel this jerky movement may predispose the repair to rupture.

Our observations and experience lead us to believe that half a fist of true active movement provides enough smooth normal profundus gliding, and that risky full fist flexion is not required. FFPH can result in buckling of the tendon followed by jerking into flexion. We therefore prefer up to half a fist of true active movement in our rehabilitation. It provides excellent results.^2,5^ We recommend that only half a fist of passive flexion be performed if therapists use place and hold.

The videos also demonstrate 5 clear examples of vented A4 pulleys with no clinically significant bowstringing with full fist flexion on the operating table. Those patients also had no clinically significant bowstringing postoperatively.
